# Impact of a statewide computed tomography scan educational campaign on radiation dose and repeat CT scan rates for transferred injured children

**DOI:** 10.1017/cts.2021.793

**Published:** 2021-05-24

**Authors:** Rosemary Nabaweesi, Chary Akmyradov, Mary E. Aitken, Phillip J. Kenney, Raghu H. Ramakrishnaiah

**Affiliations:** 1 University of Arkansas for Medical Sciences, College of Medicine (COM), Pediatrics, Little Rock, AR, USA; 2 Arkansas Children’s Research Institute, Little Rock, AR, USA; 3 University of Arkansas for Medical Sciences, COM, Biostatistics, Little Rock, AR, USA; 4 University of Arkansas for Medical Sciences, COM, Radiology, Little Rock, AR, USA; 5 University of Arkansas for Medical Sciences, COM, Pediatric Radiology, Little Rock, AR, USA

**Keywords:** Pediatric computed tomography scan protocols, radiation safety, educational campaign, radiation dose, evaluation

## Abstract

**Purpose:**

Research demonstrates that children receive twice as much medical radiation from Computed Tomography (CT) scans performed at non-pediatric facilities as equivalent CTs performed at pediatric trauma centers (PTCs). In 2014, AFMC outreach staff educated Emergency Department (ED) staff on appropriate CT imaging utilization to reduce unnecessary medical radiation exposure. We set out to determine the educational campaign’s impact on injured children received radiation dose.

**Methods:**

All injured children who underwent CT imaging and were transferred to a Level I PTC during 2010 to 2013 (pre-campaign) and 2015 (post-campaign) were reviewed. Patient demographics, mode of transportation, ED length of stay, scanned body region, injury severity score, and trauma center level were analyzed. Median effective radiation dose (ERD) controlled for each variable, pre-campaign and post-campaign, was compared using Wilcoxon rank sum test.

**Results:**

Three hundred eighty-five children under 17 years were transferred from 45 and 48 hospitals, pre- and post-campaign. Most (43%) transferring hospitals were urban or critical access hospitals (30%). Pre- and post-campaign patient demographics were similar. We analyzed 482 and 398 CT scans pre- and post-campaign. Overall, median ERD significantly decreased from 3.80 to 2.80. Abdominal CT scan ERD declined significantly from 7.2 to 4.13 (*P*-value 0.03). Head CT scan ERD declined from 3.27 to 2.45 (*P*-value < 0.0001).

**Conclusion:**

A statewide, CT scan educational campaign contributed to ERD decline (lower dose scans and fewer repeat scans) among transferred injured children seen at PTCs. State-level interventions are feasible and can be effective in changing radiology provider practices.

## Background

Excessive medical radiation in the USA, particularly to children, was highlighted in 2001 [1,2]. Between 1998 and 2005, Computerized Tomography (CT) scan utilization grew at a rate of 10% annually [1,3–5]. Among children, the rise in CT utilization was due to CT scan diagnostic efficacy, increased traumatic brain injury awareness, increased Emergency Department (ED) visits particularly following sports injuries, and patient/provider demand [4,6]. Great strides have been made since the Alliance for Radiation Safety in Pediatric Imaging (The Alliance) made radiation safety a priority for children [7,8]. The Alliance created adult and pediatric radiation protocols through collaboration with government, non-governmental agencies, vendors, and manufacturers of CT equipment [7].

The American Association of Physicists in Medicine (AAPM) Task Group designed the national guidelines on radiopharmaceutical dose for children [5]. Children’s hospitals including pediatric trauma centers (PTCs) have adopted the pediatric CT scan protocols using minimal radiation doses with optimal image quality [9–12]. However, 90% of pediatric emergency room CT scans are done in adult-focused hospitals [13,14]. Hospitals, especially those in the poor rural states, may not have the financial resources to update their CT scan equipment on a regular enough basis to keep up with manufacturers’ updates on in-built protocols and optimal functioning. Additionally, there is a lack of standardized CT equipment nomenclature used by radiologists, physicists, and CT manufacturers [15].

External change agents, such as Agency for Healthcare Research and Quality and Institute for Healthcare Improvement, may be effective at introducing innovation into healthcare systems to produce desirable, direct, and anticipated quality improvement [16]. These external change agents can play an essential role in healthcare organizational change efforts particularly in multifaceted interventions. One such agent in our state is AFMC, a not-for-profit organization whose mission is to help healthcare providers deliver the best quality of care at the lowest cost and empower patients to take control of their (families’) health.

## Intervention

In 2013, AFMC conducted a statewide educational campaign targeting CT scan utilization across all Arkansas EDs including the Arkansas Trauma System. Improvement team (advisors) conducted outreach visits to EDs using CT scan educational brochures [17] to promote CT scan appropriateness guidelines set by the American College of Emergency Physicians (ACEP), the American College of Radiology (ACR), and the Joint Commission. ED providers (faculty and staff) were educated on appropriate CT imaging utilization to reduce unnecessary medical radiation exposure, especially to children. The imaging protocol focused on radiation exposure awareness, cumulative radiation dose, and patients’ rights of imaging [4,18,19]. Additionally, AFMC outreach staff educated providers about following recommendations: (i) routinely check medical records for previous imaging studies; (ii) routinely question patients about other imaging workups; and (iii) remember radiation dose is cumulative, given that significant radiation doses to children are associated with an increased lifetime risk of cancer [17]. ED providers were educated about the five patients’ rights of imaging: (1) the right study; (2) the right order; (3) the right way; with (4) the right report; and (5) the right action. The patients’ rights incorporate the concepts of image overuse, underuse, and misuse. Implementation of patients’ rights is a difficult, complicated task that requires communication, cooperation, and collaboration among ED physicians, radiologists, radiology technologists, physicists, and trauma surgeons.

AFMC Quality Improvement team used CT scan practice guidelines from Joint Commission, American College of Radiology and the FDA to develop laminated brochures describing appropriate CT scan use, procedures to avoid unnecessary CT scans and radiation, and the risks associated with over use for providers. A variation of laminated patient CT educational brochures was availed to EDs for patient education and distribution using teach back. Patient families were also educated about the importance of providing imaging history to minimize cumulative radiation risk for cancer. Clinician providers were educated about strategies for CT scan utilization success using CT scan protocols developed by the American College of Radiology, ACEP, and Joint Commission. The protocol guidelines are summarized in Table 1.

**Table 1.** Summary of CT scan guidelines clinicians received during educational campaign

1. Develop pediatric-specific protocols for imaging with a focus on minimal dose necessary to obtain a quality image.
2. Image with radiation only when medically necessary. When ordered appropriately, the risk to benefit medical imaging is excellent. Use alternative modalities (ultrasound or MRI) when appropriate.
3. Scan only the affected region. Develop protocols for follow-up examinations (e.g., follow-up of an incidental lung nodule does not require a full chest CT).
4. Scan once only. Multiphase scans are rarely indicated in children.
5. Involve clinical staff in quality improvement initiatives relative to ordering of medical imaging studies.
6. Arkansas Children’s Hospital has developed pediatric-specific imaging protocols. Contact information for pediatric CT Technologist/Radiology Technologist was made available
7. Reduce radiation exposure during CT (children)

The goal of this study is to determine the impact of this statewide CT scan educational campaign conducted to educate clinician providers about appropriate CT scan utilization, cumulative radiation risk, and the importance of eliciting imaging history. We studied CT scans’ effective radiation dose (ERD) and repeat rates among injured children transferred to a level I PTC before and after educational campaign.

## Methods

### Data Sources

We used a data set compiled for our two previous publications evaluating the impact of a Web-Based Image repository and comparing the ERD that children received at PTC vs adult trauma centers (ATCs). We conducted a cross-sectional study using administrative data from the pediatric trauma registry and clinical data from the Picture Archiving Communication System (PACS). All children who met the state trauma system’s definition of a traumatic injury (trauma team activation, penetrating injuries, and ED deaths) were included in the registry. The only Level I PTC in the state houses the trauma registry. The PTC receives injured children from 64 accredited trauma system facilities that utilize a centralized web-based image repository. A dedicated trauma team manages the pediatric trauma registry to ensure timeliness, accuracy, and data completeness.

The Arkansas state trauma system is composed of trauma enters, the trauma call center, and the Trauma Advisory Council. At baseline, there were 64 trauma centers, 6 of these were level Is, 5 level IIs, 18 Level IIIs, and 35 level IVs [13], serving 62 EDs. The system includes two PTCs and one burn center. As of March 12, 2019, our trauma system composed of 56 trauma centers, two level Is, five level IIs, sixteen level IIIs, and thirty-three level IVs. Four out of state, level I trauma centers were excluded from this analysis. Since 2015 (post-campaign period), we have had more hospitals receive the state’s trauma designation including, one level II, two level IIIs, and 8 level IVs.

### Patient and Variable Selection

All injured children under 17 years of age who met the trauma criteria, underwent CT imaging, and were transferred to the PTC from an outlying hospital during calendar year 2010, 2011, 2012, 2013 (pre-campaign), and 2015 (post-intervention) were included. AFMC outreach staff conducted the education campaign in 2014. We assigned radiographic studies to one of the two categories defined here, outside or in-house studies. Outside studies were defined as CT imaging performed at an ATC and transmitted to the PACS. In-house studies were CT imaging performed at the PTC. All children transferred to the PTC arrived in the ED where additional imaging, if required, is typically performed before the child is discharged to intensive care unit, operating room (OR), admitting floor, or home.

Independent patient level factors studied as potential covariates included age, race, gender, mode of transportation, Injury Severity Score (ISS), and ED disposition. The independent hospital level factor was the transferring facility Trauma Center level. Hospital level factors studied included the region of the state, hospital type (urban, rural, critical care access, or community), hospital size (large, medium, and small), and location of radiology services (in house or out sourced). Hospital size was based on the Healthcare Utilization Project definition and was nested in location and teaching status [20]. Our primary outcomes of interest were CT scan ERD and repeat rate.

### Statistical Analysis

We summarized data using frequency and percentages for categorical variables and median (interquartile range) for continuous variables. We matched scans done in ATC and PTC by body regions for each patient. Effective radiation dosage was calculated for each scan with DLP and age information [21]. Scans that did not have radiation dosage were excluded. ERD was stratified by body region (head, neck, chest, and abdomen), race, age, gender, transportation, ED disposition, ISS [22], and trauma center level. Median ERD was compared between pre-campaign and post-campaign observations using the Wilcoxon Rank Sum Test. ISS was categorized by convention as mild (1–8), moderate (9–16), severe (17–25), or life threatening (>26). *P* values less than or equal to 0.05 were considered to be statistically significant. Statistical analysis was performed using the SAS™ software version 9.4 (SAS Institute Inc., Cary, NC, USA). The study was approved by the University’s Institutional Review Board.

## Results

**Fig. 1.**
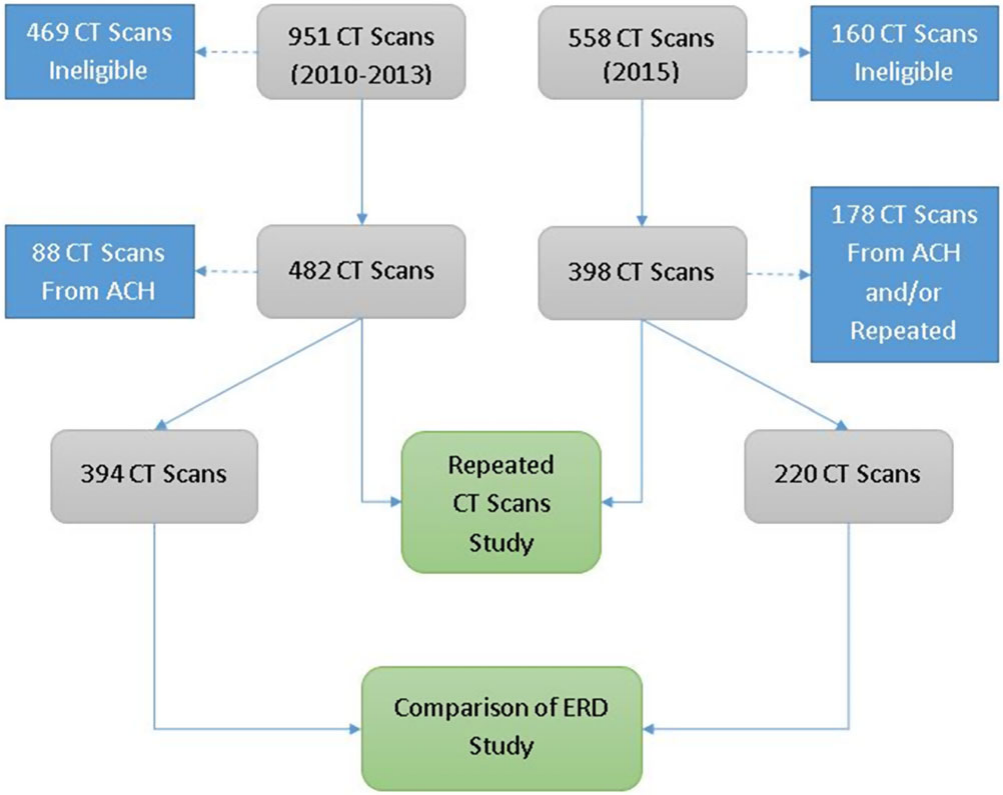
Data flow diagram.

Data were available on 251 injured children from 2010 to 2013 (pre-campaign) and 134 injured children during 2015 (post-campaign) who were transferred to a Level I pediatric center from ATCs within our state. There was a total of 45 and 48 ATCs transferring children during pre-campaign and post-campaign, respectively. Children who received a CT scan of the abdomen, chest, head, or neck were included in the study. The PACS had 951 and 558 CT scans in the pre- and post-campaign periods, respectively. During the pre-campaign period, 482 (50.7%) CT scans were eligible for the study, while 398 (71.3%) CT scans were eligible post-campaign. To assess the impact of the educational campaign on ERD, CT scans done at the Level I PTC and/or repeated CT scans at the PTC were eliminated because our previous published study [12] demonstrated that PTC ERD was lower than ACR and AAPM recommended pediatric doses. The final data set composed of 394 CT scans during pre-campaign and 220 CT scans during the post-campaign periods as displayed in Fig. 1.

For both periods, most hospitals are located in the Arkansas Valley and South West regions (Table 2). Of the 45 and 48 transferring hospitals assessed during the pre-/post-campaign periods, majority (44% and 42%, respectively) were urban, followed by rural (29%) and critical area hospitals (33%). Large hospitals [21] comprised the majority of transferring hospitals. Among the known radiological services, most CT scans were taken in house and most children were transferred from level IV Trauma centers. Demographic characteristics were comparable across pre-post campaign periods. In the post-campaign period, majority of children in the study were white (83.6%), male (61%), and had a median injury severity score of 9.0 (Table 3). While patient demographics were similar between pre- and post-campaigns, ground transportation increased from 60% to 79%, median ED length of stay increased from 3.6 h to 4.1 h, ED disposition to ICU/OR decreased from 30.3% to 23.1%.

**Table 2.** Hospital characteristics (N = 93 hospitals)

	Pre-campaign	Post-campaign
Total	45	48
Region of state		
Arkansas Valley	7 (15.56%)	9 (18.75%)
Metropolitan	6 (13.33%)	7 (14.58%)
North Central	8 (17.78%)	5 (10.42%)
North East	5 (11.11%)	5 (10.42%)
North West	6 (13.33%)	5 (10.42%)
South East	5 (11.11%)	8 (16.67%)
South West	8 (17.78%)	9 (18.75%)
Hospital type		
Critical	12 (26.67%)	16 (33.33%)
Rural	13 (28.89%)	12 (25.00%)
Urban	20 (44.44%)	20 (41.67%)
Hospital size*		
Large	21 (46.67%)	20 (41.67%)
Medium	10 (22.22%)	9 (18.75%)
Small	14 (31.11%)	19 (39.58%)
Radiology services		
Both	4 (8.89%)	3 (6.25%)
In-house	18 (40.00%)	17 (35.42%)
Outsourced	8 (17.78%)	12 (25.00%)
Unknown	15 (33.33%)	16 (33.33%)
Trauma center		
Level II	3 (6.67%)	4 (8.33%)
Level III	16 (35.56%)	14 (29.17%)
Level IV	20 (44.44%)	25 (52.08%)
Not Applicable	6 (13.33%)	5 (10.42%)

* Hospital Bed Size Based on Healthcare Cost Utilization Project (HCUP) is embedded in location and teaching status.

**Table 3.** Patient characteristics (N = 385)

	Pre-campaign	Post-campaign
Total	251	134
Race		
White	202 (80.48%)	112 (83.58%)
Black	27 (10.76%)	21 (15.67%)
Hispanic	11 (4.38%)	-
Other	11 (4.38%)	1 (0.75%)
Gender		
Female	93 (37.05%)	52 (38.81%)
Male	158 (62.95%)	82 (61.19%)
Age (years), Median (IQR*)	6.65 (2.2, 12.7)	8.50 (4.0, 13.0)
Transportation		
Air	100 (39.84%)	28 (20.90%)
Ground	151 (60.16%)	106 (79.10%)
ED^†^ LOS^‡^ (Hours), Median (IQR)	3.61 (2.3, 4.8)	4.11 (2.9, 5.6)
ED disposition		
ICU^§^/OR^||^	76 (30.28%)	31 (23.13%)
Other^¶^	175 (69.72%)	103 (76.87%)
Injury Severity Score, Median (IQR)	9.00 (5.0, 14.0)	9.00 (5.0, 14.0)

* Interquartile range.

† Emergency Department.

‡ Length of Stay.

§ Intensive Care Unit.

|| Operating Room.

¶ Floor and Home.

Following the statewide CT scan educational campaign the overall, median ERD at transferring hospitals significantly decreased from 3.80 to 2.80 (Table 4). Post-campaign median ERD was reduced across all independent factors except for female gender, age >15 years, children transported by air, discharged to the OR or ICU, and suffered severe or life-threatening injury. The odds of a child receiving a repeat CT scan on arrival at the PTC ED post-campaign was significantly reduced in children who arrived by air, with moderate and life-threatening injuries, transferred from Level II and Level III trauma centers and discharged to ICU, operating room, and all dispositions (Table 5).

**Table 4.** Children’s effective radiation dose when scanned at transferring hospitals

	Pre-campaign		Post-campaign		*P*-value
	N	Median (Q1, Q3)	N	Median (Q1, Q3)	
Total	394	3.80 (2.47, 5.84)	220	2.80 (1.62, 4.37)	<.0001
Race					
White	318	3.83 (2.55, 5.96)	189	2.95 (1.63, 4.60)	0.0001
Black	44	4.01 (2.02, 5.20)	27	2.73 (1.41, 3.67)	0.0044
Hispanic	19	3.86 (1.56, 7.39)		-	
Other	13	2.89 (2.04, 4.98)	4	3.31 (1.51, 4.70)	NS
Age (years)					
<1	23	3.91 (2.73, 6.89)		-	
1–4	79	3.33 (2.07, 5.00)	15	2.27 (0.78, 4.32)	0.0147
5–9	108	3.28 (1.76, 5.17)	63	2.38 (1.45, 3.83)	0.0113
10–14	143	4.14 (3.10, 7.20)	98	3.07 (1.62, 4.43)	<.0001
>15	41	3.13 (2.49, 6.98)	44	3.44 (2.19, 9.26)	NS
Gender					
Female	144	3.39 (2.24, 5.14)	99	3.10 (1.79, 5.79)	NS
Male	250	4.02 (2.60, 6.01)	121	2.74 (1.60, 4.25)	<.0001
Transportation					
Air	163	3.79 (2.31, 6.89)	48	3.37 (1.96, 5.52)	NS
Ground	231	3.85 (2.63, 5.47)	172	2.76 (1.61, 4.21)	<.0001
ED* Disposition					
ICU^†^/OR^‡^	118	3.44 (2.49, 6.43)	55	3.06 (1.61, 6.62)	NS
Other^§^	276	3.90 (2.39, 5.67)	165	2.76 (1.62, 4.25)	<.0001
CT^||^ Body Region					
Abdomen	83	7.20 (4.47, 11.54)	28	4.13 (2.83, 7.81)	0.0263
Chest	21	7.39 (4.77, 10.00)	37	6.62 (3.95, 10.41)	NS
Head	203	3.27 (2.47, 4.59)	105	2.45 (1.61, 3.41)	<.0001
Neck	87	3.25 (1.52, 4.64)	50	2.19 (0.87, 4.09)	0.0329
Injury Severity Score					
Mild	139	3.91 (2.21, 6.56)	89	2.76 (1.84, 4.29)	0.0011
Moderate	185	3.78 (2.44, 5.30)	88	2.71 (1.62, 4.17)	0.0009
Severe	61	3.38 (2.55, 6.52)	35	2.95 (1.43, 8.24)	NS
Life threatening	9	4.63 (3.23, 7.49)	7	4.31 (2.78, 21.71)	NS
Trauma Center					
Level 2	67	3.49 (2.48, 5.09)	33	2.76 (2.16, 4.60)	NS
Level 3	219	3.86 (2.30, 5.84)	109	2.95 (1.73, 4.25)	0.0023
Level 4	94	3.96 (2.48, 6.68)	66	2.93 (1.51, 4.38)	0.0048
Not Applicable	14	3.84 (2.91, 9.77)	12	2.28 (1.61, 4.75)	NS

* Emergency Department.

† Intensive Care Unit.

‡ Operating Room.

§ Floor and Home.

|| Computed Tomography.

**Table 5.** Frequencies and odds ratios of repeated CT scans at pediatric trauma center

	Pre-campaign		Post-campaign		Odds ratio (95% CI)	*P*-value
	Non-repeated	Repeated	Non-repeated	Repeated		
Transportation						
Air	104	59 (36%)	105	17 (14%)	0.29 (0.16, 0.52)	<.0001
Ground	191	40 (17%)	243	33 (12%)	0.65 (0.39, 1.07)	NS
ED* Disposition						
ICU^†^/OR^‡^	62	56 (47%)	95	24 (20%)	0.28 (0.16, 0.50)	<.0001
Other^§^	233	43 (16%)	253	26 (9%)	0.56 (0.33, 0.94)	0.0269
ISS^||^						
Mild	124	15 (11%)	147	11 (7%)	0.62 (0.27, 1.40)	NS
Moderate	128	57 (31%)	131	20 (13%)	0.34 (0.19, 0.60)	0.0002
Severe	41	20 (33%)	54	14 (21%)	0.53 (0.24, 1.18)	NS
Life Threatening	2	7 (78%)	15	5 (25%)	0.10 (0.01, 0.62)	0.0137
Trauma Center						
Level 2	50	17 (25%)	55	3 (5%)	0.16 (0.04, 0.58)	0.0053
Level 3	163	56 (26%)	149	24 (14%)	0.47 (0.28, 0.79)	0.0049
Level 4	73	21 (22%)	117	20 (15%)	0.59 (0.30, 1.17)	NS
Not Applicable	9	5 (36%)	27	3 (10%)	0.20 (0.04, 1.01)	NS

* Emergency Department.

† Intensive Care Unit.

‡ Operating Room.

§ Floor and Home.

|| Injury Severity Score.

## Discussion

We sought to evaluate the impact of a statewide CT scan educational campaign in a low-resourced rural southern state. We determined that Medicare-funded educational intervention contributed to children receiving lower ERDs from CT scans and experienced markedly reduced rates of repeat CT scans once they arrived at the level I PTC. There are not many studies published on statewide academic detailing for the As Low as Reasonably Allowable (ALARA) campaign. Most studies evaluate educational interventions conducted at single institutions or healthcare systems. Fernandes and colleagues evaluated the impact of an Image Wisely and Image Gently campaign on adult and pediatric CT scans across a multihospital healthcare system [3] and demonstrated results similar to ours for their pediatric population. In addition to a reduction in radiation dose per study, their campaign resulted in a reduction in CT utilization and a compensatory rise in ultrasound use.

The pre-post radiation dose difference among older children was not significant perhaps because the body habitus of older children resembles the body habitus of the adult.

### Limitations

We conducted a retrospective analysis limited to the merged clinical and administrative data sets. Tapping into hospital association registries could potentially reveal factors illustrating facility infrastructure variability and provide us more granular results. The retrospective analysis did not allow us to account for externalities such as providers accessing ALARA principles on their own and the impact of a concurrently implemented web-based image repository.

We did not study mechanisms of action that caused the change in radiation dose, and since we did not conduct a pretest and post-test, we are not on solid footing in attributing our findings to the educational intervention. For instance, we did not take into consideration the effect of parents refusing CT scans for their children.

Another limitation of the study was not taking into account CT scan inventory to determine the age, manufacturer, and number of equipment available to small and large trauma centers. This could account for varying results across geographic regions and hospital sizes.

Similar to our previously published studies [9,12], we did not identify clinically indicated repeat CT scans to exclude them from unwarranted repeated CTs. The failure to exclude clinically indicated repeat scans may have conservatively biased our findings toward a weaker campaign impact.

## Conclusion

A statewide educational campaign contributed to the ERD decline among injured children transferred to a PTC. This indicates that state-level interventions are feasible and can be effective and that further interventions/studies using audit feedback, education, and policy change are warranted.
